# Simultaneous sEMG Classification of Hand/Wrist Gestures and Forces

**DOI:** 10.3389/fnbot.2019.00042

**Published:** 2019-06-19

**Authors:** Francesca Leone, Cosimo Gentile, Anna Lisa Ciancio, Emanuele Gruppioni, Angelo Davalli, Rinaldo Sacchetti, Eugenio Guglielmelli, Loredana Zollo

**Affiliations:** ^1^Unit of Biomedical Robotics and Biomicrosystems, Universiã Bio-Medico di Roma, Rome, Italy; ^2^Italian Workers' Compensation Authority (INAIL), Vigorso di Budrio, Bologna, Italy

**Keywords:** pattern recognition, surface electromyography, hand gestures recognition, prostheses, gestures classifier, force classifiers, non-linear logistic regression, linear discriminant analysis

## Abstract

Surface electromyography (sEMG) signals represent a promising approach for decoding the motor intention of amputees to control a multifunctional prosthetic hand in a non-invasive way. Several approaches based on proportional amplitude methods or simple thresholds on sEMG signals have been proposed to control a single degree of freedom at time, without the possibility of increasing the number of controllable multiple DoFs in a natural manner. Myoelectric control based on PR techniques have been introduced to add multiple DoFs by keeping low the number of electrodes and allowing the discrimination of different muscular patterns for each class of motion. However, the use of PR algorithms to simultaneously decode both gestures and forces has never been studied deeply. This paper introduces a hierarchical classification approach with the aim to assess the desired hand/wrist gestures, as well as the desired force levels to exert during grasping tasks. A Finite State Machine was introduced to manage and coordinate three classifiers based on the Non-Linear Logistic Regression algorithm. The classification architecture was evaluated across 31 healthy subjects. The “hand/wrist gestures classifier,” introduced for the discrimination of seven hand/wrist gestures, presented a mean classification accuracy of 98.78%, while the “Spherical and Tip force classifier,” created for the identification of three force levels, reached an average accuracy of 98.80 and 96.09%, respectively. These results were confirmed by Linear Discriminant Analysis (LDA) with time domain features extraction, considered as ground truth for the final validation of the performed analysis. A Wilcoxon Signed-Rank test was carried out for the statistical analysis of comparison between NLR and LDA and statistical significance was considered at *p* < 0.05. The comparative analysis reports not statistically significant differences in terms of F1Score performance between NLR and LDA. Thus, this study reveals that the use of non-linear classification algorithm, as NLR, is as much suitable as the benchmark LDA classifier for implementing an EMG pattern recognition system, able both to decode hand/wrist gestures and to associate different performed force levels to grasping actions.

## 1. Introduction

The use of surface electromyography (sEMG) allows the non-invasive extraction of pattern information useful to control active prosthetic hands. In the last 70 years, several solutions have been proposed to extract gestures information from sEMG (Ciancio et al., 2016, 2017); the most simple were based on on-off (Scott and Parker, 1988), on Agonist/Antagonist (Popov, 1965) and Proportional Control (Fougner et al., 2012). Targeted Muscle Reinnervation (TMR) enabled amputees with shoulder disarticulation or transhumeral amputation to control motorized prosthetic devices with multi-DoFs (Aszmann et al., 2015) in a natural way. Pattern recognition methods enabled performance improvements to reach an intuitive and coordinated control (Li et al., 2018). Moreover, these techniques allowed the increasing of the number of controllable Degree of Freedoms (DoFs) (Ciancio et al., 2016). Different classification algorithms have been proposed in literature, including Euclidean Distance, Non-Linear Logistic Regression, k-Nearest Neighbors (kNN), Hidden Markov Model (HMM), Artificial Neural Network (ANN), Support Vector Machine (SVM), Linear Discriminant Analysis (LDA) (Chowdhury et al., 2013). However, different arm positions (Geng et al., 2012), electrode shift (Young et al., 2012a), signal non-stationarity (Lorrain et al., 2011) and force variation (Scheme and Englehart, 2011) can affect the pattern-recognition accuracy and robustness. In addition, physiological factors as motor unit (MU) recruitment, MU firing rate and contraction type (e.g., isometric, isotonic, concentric, or eccentric) make difficult the extraction of sEMG-force relationship due to non-linear factors (Farina et al., 2007; Disselhorst-Klug et al., 2009; Staudenmann et al., 2009).

In literature two main approaches have been proposed to find a relationship between muscular activation and force: mathematical models and machine learning techniques. Force estimation, based on surface electromyographic measurements, was determined through a sEMG-force mathematical relationship, by applying Non-linear Wiener Hammerstein (NLWH) and Spectral Analysis Frequency Dependent Resolution (SPAFDR) models (Potluri et al., 2013). Buchanan et al. (2004) presented a computational neuro musculoskeletal model of the human arm with the aim to estimate muscle forces, joint moments and joint kinematics from neural signals. Moreover, “crosstalk risk factors” (CRF), as the dependency of the relationship between the sEMG signals, muscle length and isometric contraction force, had to be quantified to understand the effectiveness of the muscular co-ordination in generating force (Disselhorst-Klug et al., 2009).

Related to machine learning techniques, Srinivasan et al. (2012) proposed a method for estimating forces from surface electromyography (sEMG) signals with Artificial Neural Network (ANN). Wu et al. (2017) proposed a force estimation method employing a Regression Neural Network (GRNN) trained with sEMG and force signals. In the most recent study, Ren et al. (2017) divided force signals in different grades from 0 N to 16 N, expressed as percentage of the Maximum Voluntary Contraction (MVC). They used SVM to establish non-linear regression relationship between sEMG and force. Lv et al. (2017) used Linear Discriminant Analysis (LDA) to classify five finger gestures at two different levels of force (i.e., 10% MVC and 50% MVC), by using EMG and accelerometer signals. Li et al. (2018) proposed a method based on deep neural network to derive sEMG-force regression model for force prediction at eight different force levels. Al-Timemy et al. (2016) reported that force level variations negatively affected the performance of PR system and caused the increase of the classification error rates. However, an increasing of 6−8% in the classification performance can be reached by applying Time-Dependent Power Spectrum Descriptors (TD-PSD) features extraction to four classifiers [ i.e., LDA, Random Forest (RF), Naive Bayes (NB), k-Nearest Neighbor (kNN)] and training with all forces across nine trans-radial amputees. In order to investigate the performance of PR system in presence of variations in force, Scheme and Englehart (2011) evaluated a LDA classifier with Time-Domain (TD) features extraction, by using data of 10 classes performed at 20% and 80% of the strongest and reproducible contraction, except for the tenth class of no motion. The LDA classifier performed an error rate equals to 17% when trained and tested using data of 11 healthy subjects at all force levels. The error increased at 31−44% when trained at one force level and tested with all force levels. Subsequently, the effect of contraction strength on pattern recognition based control was studied in Scheme and Englehart (2013). By using a LDA classifier trained with dynamic ramp data of 10 healthy subjects, the classification error significantly improved (11.16±0.54%).

Different strategies have been developed by combining the above techniques to make the control most fluid and intuitive for the user. Two proportional control algorithms were used to obtain a robust and proportional velocity commands that could improve the usability of PR (pattern recognition) based control (Scheme et al., 2014). Fougner et al. (2014) presented a novel pattern recognition system with mutex on-off control or proportional control of a commercial prosthetic hand and wrist. In Young et al. (2012b) three classification strategies were introduced and compared in order to provide simultaneous DoFs control. The first classification approach used a single linear discriminant analysis (LDA) classifier to discriminate both discrete and combined motions. All the discrete and combined gestures were considered as separated classes. The second proposed approach was based on a hierarchical classification strategy and consisted of a hierarchy of LDA classifiers. The highest classifier in the hierarchy determined a motion class for a single DoF by using both discrete and combined motion data. The output of this classifier determined which classifier of the second level could be used for discriminating the motion class of a second DoF. Finally, the parallel classification strategy employed one LDA classifier for each DoF and the decision of the single classifier is independently defined. The parallel classification strategy was presented also to either allow the simultaneous control of three-digits of a monkey (Baker et al., 2010) or to control the elbow and hand/wrist movement of an active myoelectric transhumeral prosthesis (Boschmann et al., 2011). No hierarchical strategy has ever been proposed to simultaneously identify desired gestures and forces.

This work aims at proposing and testing a hierarchical pattern recognition strategy to contemporary identify desired hand/wrist gestures and force levels. In details, a Finite State Machine (FSM) scheme (Figure 2) is introduced to manage desired hand/wrist gestures and force levels, following a hierarchical approach.

Differently from Young et al. (2012b) the hierarchical classification system is used to discriminate simultaneously hand/wrist gestures and desired force levels. In details, the highest NLR classifier, i.e., the “hand/wrist gestures classifier,” is devoted to identify the desired hand/wrist class among seven gestures. The output of this classifier determines the next classifier used in the hierarchy. If the output of this classifier is “Spherical” motion class, then the “Spherical force classifier” is used to determine the desired force level to exert on an object. This second classifier is conditioned on the decision of the first classifier. The same strategy is adopted if the output of the first classifier is “Tip” motion class. In this case, the “Tip force classifier” lower in the hierarchy is used to determine the desired force levels. Thus, the classifiers of the second level of the hierarchy discriminate the force levels applied during the related grasping class. If, instead, the output of the first classifier is any hand/wrist gestures different from “Spherical” or “Tip” gesture, no force classifiers are activated. Hand and wrist gestures are classified by using the single “hand/wrist gestures classifier.” When the “Spherical” or “Tip” state is chosen, the “Spherical force classifier” or the “Tip force classifier” is respectively activated in a hierarchical way to discriminate between three different force levels (i.e., Low, Medium, and High). The FSM use allowed the two classifiers of different grades of the hierarchy to work simultaneously. Until the “Spherical” or “Tip” state is classified by “hand/wrist gestures classifier,” the “Spherical force classifier” or the “Tip force classifier” intervenes to discriminate force levels.

The NLR and LDA algorithms are employed for implementing the hierarchical classification approach, since LDA and NLR retained statistically similar value for F1Score performance and computational burden, despite LDA has the fewest number of classification parameters (Bellingegni et al., 2017). The number of gestures is increased from five (Bellingegni et al., 2017) to seven and the classification is extended to three different force levels, by using the same number of sensors. Force information is provided only for the two grasping classes (i.e., “Spherical” and “Tip”) in which an object interaction is expected. Seven hand/wrist gestures (i.e., Rest, Spherical, Tip, Platform, Point, Wrist supination, and Wrist pronation) had been discriminated by using a Non-linear Logistic Regression (NLR) algorithm. When the “Spherical” or the “Tip” class are identified, a second NLR-algorithm-based classifier, i.e., respectively, “Spherical force classifier” or “Tip force classifier” is activated simultaneously in order to discriminate three force levels (i.e., Low, Medium, and High).

The same hierarchical pattern recognition strategy was implemented with three linear classifiers (“hand/wrist gestures classifier,” “Spherical force classifier” and “Tip force classifier”), based on LDA with time domain features extraction. The performance of each algorithm (NLR and LDA) were measured by means of F1Score value and statistical analysis had been based on the Wilcoxon Signed-Rank test, which had been shown to be appropriate for comparing different classifiers in common datasets (Demšar, 2006). A comparative analysis among NLR and LDA with features extraction was implemented in order to define the most suitable classification algorithm for the realization of a gestures and forces classification architecture to control of a prosthetic device. Thus, in literature, several studies have been considered the LDA classifier with features extraction as ground truth (Simon et al., 2011; Young et al., 2014) and it can be used for the online control of prosthetic devices (Resnik et al., 2017) that is commercially available by COAPT (https://www.coaptengineering.com).

The performance of the proposed approach are evaluated during an experimental session involved 31 healthy subjects. The users are asked to perform seven hand/wrist motions and to replicate three different force levels during the “Spherical” and “Tip” grasps.

The paper is organized as follows. Section II describes the proposed force/gesture classification approach and the experimental setup used to collect sEMG and force data. Section III reports the results in terms of F1Score and accuracy of each classifier trained with both NLR and LDA algorithms. Section IV discusses the achieved results and then it reports the comparative analysis among NLR and LDA classifiers in terms of F1Score. The last section draws the conclusion, including some considerations regarding the comparative analysis between NLR and the LDA benchmark classifier, limits and future works.

## 2. Materials and Methods

### 2.1. Forces/Gestures Classification Approach

**Figure 1:**
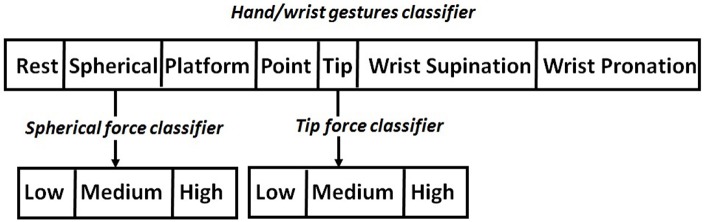
Hierarchical classification strategy. “Hand/wrist gestures classifier” allowed the identification of the desired motion class among seven different gestures. “Tip force classifier,” lower in the hierarchy, allowed the classification of three force levels for “Tip” gesture. “Spherical force classifier,” lower in the hierarchy, allowed the classification of three force levels for “Spherical” gesture.

A hierarchical pattern recognition strategy was proposed for the classification of the desired hand/wrist gestures and force levels from muscular signals (Figure 1). The FSM coordinated the hierarchical activation of the three classifiers implemented both with NLR and LDA algorithms for doing a comparison in terms of F1Score performance.The highest classifier in the hierarchy was a single classifier able to discriminate seven discrete hand/wrist motion classes. The output of this classifier determined the desired hand/wrist gesture and, in case of “Spherical” or “Tip” class, the force classifier, lower in the hierarchy, to be activated. Thus, the force classifiers were activated for force levels recognition.

The described hierarchy was implemented adopting NLR algorithm for both gesture and force classifiers. The same hierarchy was then reproduced using LDA algorithm in order to perform a comparative analysis. The Linear Discriminant Analysis (LDA), using time domain of the EMG signal, was frequently employed in literature because it was considered an efficient algorithm, simple to train and with an optimal compromise in terms of computational burden (Young et al., 2014). The Wilcoxon Signed-Rank test applied to the F1Score values was performed with significance threshold set to 0.05.

The FSM coordinated the three classifiers activation (i.e., one for hand/wrist gestures and two for force levels). The FSM approach was characterized by the following key features:

- The FSM can only be in a fixed set of states.
- The FSM can only be in one state at a time.
- A sequence of inputs was sent to the FSM.

**Figure 2:**
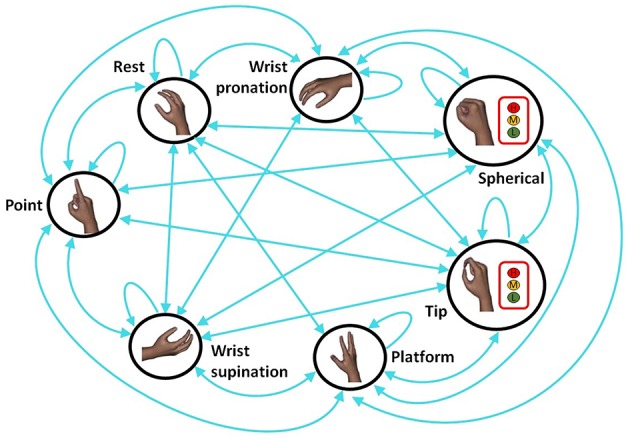
Finite State Machine (FSM) strategy for the classification of seven different hand/wrist gestures and three force levels: the blue circle states indicated the hand gestures and wrist motions and they were all classified through the “hand/wrist gestures classifier.” Three force levels (Low, Medium, and High) can be classified through the “Spherical or Tip force classifier” if the “hand/wrist gestures classifier” discriminated respectively the “Spherical” or “Tip” state. If the “Spherical” or “Tip” state was classified, the hierarchical classification strategy was adopted.

The proposed classification system was characterized by three different classifiers (Figure 2):

- The “hand/wrist gestures classifier” was able to discriminate seven states, corresponding to seven hand and wrist gestures (blue circle states in Figure 2). This classifier was always active and it was the highest classifier in the hierarchy (Figure 2).
- The “Spherical force classifier” was able to discriminate three force levels (*i.e., Low, Medium, and High Level* shown in Figure 2 in the red box). It was active if the “Spherical" gesture was identified and it was lowest in the hierarchy (Figure 2).
- The “Tip force classifier” was able to discriminate three force levels (*i.e., Low, Medium, and High Level* shown in Figure 2 in the red box). It was active if the “Tip" gesture was identified and it was lowest in the hierarchy (Figure 2).

FSM determined the following different scenarios: a single classification approach used “hand/wrist gestures classifier” to recognize seven discrete hand/wrist motion classes; the classification approach become hierarchical when the output of this classifier was the “Spherical” or “Tip” motion class. In this case, a second classifier (force classifier) was activated. Until the FSM system remained in one of these two states (i.e., “Spherical” or “Tip”), the output of the FSM system provided hand/wrist gestures and the force levels information. Otherwise, if the FSM system was in a different state from the “Spherical” or “Tip,” only the single “hand/wrist gestures classifier” was activated and the gesture information was supplied.

The force classifiers managed only a three classes classification problem related to three different force levels ( *i.e., Low, Medium, and High*).

**Figure 3:**
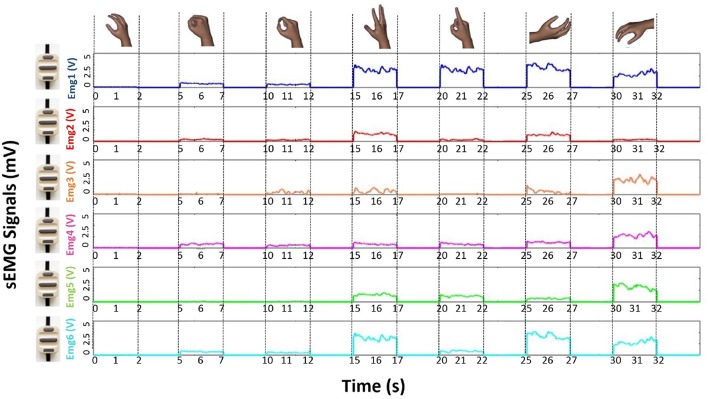
Plot of the raw sEMG recording for the six EMG channels, related to all the seven performed movements of a single acquisition session from one of the subjects who was involved into the experiment. The plot of raw sEMG recording of “Spherical” and “Tip” classes are related to muscular activations performed at medium force level.

**Figure 4:**
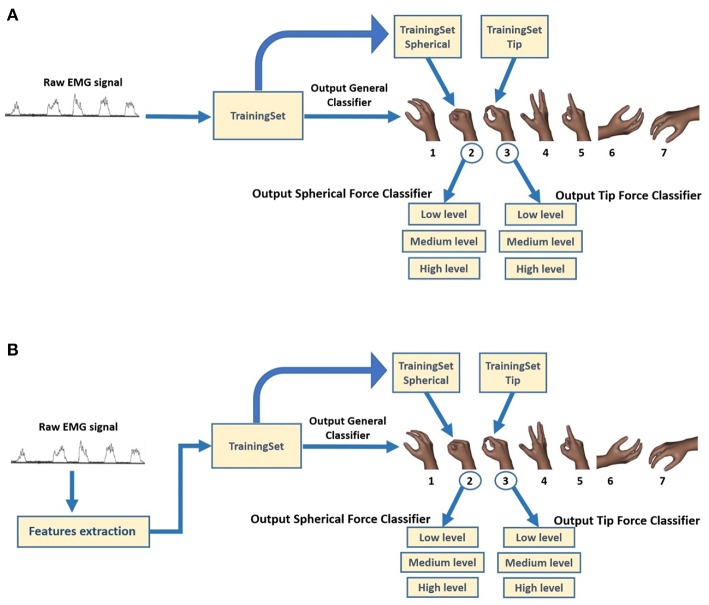
Block diagram of classification system for the creation of three different TrainingSet for obtaining the relative output classes. **(A)** For the NLR classifiers, the raw sEMG signals are used as input features in order to speed up the training and cross validation of the NLR algorithm. **(B)** For the LDA classifiers, five commonly used time domain features were extracted: Mean Absolute Value (MAV), Root Mean Square (RMS), Slope Sign Change (SSC), Waveform Length (WL), and Variance (σ^2^).

The raw sEMG recording for the six EMG channels, related to all the seven performed movements of a single acquisition session, was reported in (Figure 3). In details, the enveloped EMG signal was acquired at 1 KHz to create three Datasets, used for both the NLR and LDA algorithms (Figure 4). For the NLR classifiers, the “raw” sEMG signals were used as input features in order to speed up the training and cross validation of the NLR algorithm (Figure 4A). On the other hand, for the LDA classifiers, five commonly used time domain features were extracted: Mean Absolute Value (MAV), Root Mean Square (RMS), Slope Sign Change (SSC), Waveform Length (WL) and Variance (σ^2^) (Figure 4B). In this case, the features extraction avoided the generation of large-scale-dataset without performing the downsampling step and the time to complete the training is not too long. The TrainingSet of the “hand/wrist gestures classifier” was composed by sEMG signals related to all the seven states of FSM. This TrainingSet included the recording of Spherical and Tip gestures performed at three different force levels in order to correctly classify gestures independently from muscular contraction changes due to force variations. The TrainingSets of “Spherical and Tip force classifiers” were composed only by sEMG data expressing different muscular contraction levels for these gestures. The NLR and LDA machine learning algorithms and dataset organization are provided below.

### 2.2. NLR Classification Algorithm and Dataset Organization

In each subject's acquisition, the sEMG data were organized in a 84000 * 6 dimensions matrix. Each column of the matrix was coupled with an EMG sensor. Firstly three-way data split approach (Ripley, 2007) was applied to the dataset (84000 * 6 sEMG data) and the Training Set (TR), the Cross Validation Set (CVS) and the Test Set (TS) were set to contain 60, 20, and 20% of the data, respectively. A random shuffle was implemented for filling these subsets with a proper proportion of all classes samples distribution.

The unique operation done on sEMG data was the scaling: it consists of subtracting the mean value to each signal and dividing the result by the range, as done in Bellingegni et al. (2017). Then,downsampling (with a step = 10, 100 Hz) was applied to reduce the data dimensions and training process.

The scaled “raw” sEMG data were directly used as input for the NLR model, without performing any features extraction. The use of only “raw” sEMG signals allowed a significant reduction of the classification time and of the response time without loss of system performance (Nazarpour, 2005; Dohnalek et al., 2013; Benatti et al., 2014). Moreover, the use of “raw” scaled sEMG signals (Figure 3) as input features approximated the class evaluation time and system readiness to the sampling time (Bellingegni et al., 2017). The discarded data rising from the downsampling process (90% of initial data) composed a new set of data called Generalization Set (GS) used as a second test to obtain an estimation of the generalization capability of each classifier. The three way data split approach was applied on the data coming from downsampling process (10% of initial data): TR, CVS, and TS were set to contain 6, 2, and 2% of the data, respectively. The TR and CV were used to train and cross validate the classifiers and the TS and GS were employed to test the performance of the classifiers. In details, the TR was used to train the supervised classification algorithm by minimizing a specific cross-entropy error cost function:

(1) $$\begin{array}{l} J(\theta ,\theta_{0})=-\frac{1}{m}\left[\sum_{i=1}^{m}y^{\left(i\right)}\cdot \ln g\left(\theta^{T}\cdot x^{\left(i\right)}+\theta_{0}\right)\right]\\ \;-\frac{1}{m}\left[\sum_{i=1}^{m}\left(1-y^{\left(i\right)}\right)\cdot \ln\left(1-g\left(\theta^{T}\cdot x^{\left(i\right)}+\theta_{0}\right)\right)\right] \end{array}$$

where *m* is the number of samples of TrainingSet, *y*^(*i*)^ is the known class membership of the *i*-th sample, θ and θ_0_ are the classification parameters and *g*(·) is the logistic function. Resilient Backpropagation (RProp) was chosen as minimization algorithm (Baykal and Erkmen, 2000; Bellingegni et al., 2017) for the NLR. Each single classifier was iteratively trained with all possible configurations of its internal parameters that had an appropriate range of values (Bellingegni et al., 2017).

To avoid overfitting and explore the best model, the CV was used to evaluate the performance of classifiers for each set of internal parameters (Bellingegni et al., 2017). In this study, the goodness of the classification was evaluated in terms of F1Score because it was considering more robust, in lieu of accuracy, to assess the performance (Powers, 2011). Once the optimal classification model had been chosen, TS was used to evaluate the performance of classifier when new features were introduced as input.

The NLR algorithm calculated the class membership probability by using the following logistic function:

(2) $$P(1|x,\theta )=\{\begin{array}{l} g(\theta^{T}\cdot x)=\frac{1}{1+e^{-(\theta^{T}\cdot x+\theta_{0})}}\\ 1-P(y=0|x,\theta ) \end{array}$$

where θ and θ_0_ are, respectively, the classification parameters vector and bias term, while *g*(·) is the logistic function. Additional polynomial features (e.g., $x_{1},x_{2},x_{1}*x_{2},x_{1}^{2},x_{2}^{2}$) were introduced to make non-linear this logistic regression model. The prediction of class labels *h*_θ_ for the NLR algorithm was achieved by comparing the probability distribution *P*(*y*|*x*) with a decision threshold (TH):

(3) $$h_{\theta }=\{\begin{array}{l} P(1|x,\theta )\geq TH\to 1\\ P(1|x,\theta )<TH\to 0 \end{array}$$

### 2.3. LDA Classification Algorithm and Dataset Organization

In order to create linear classifiers able to provide accurate movement classes and force levels recognition, a proper features set needs to be chosen to represent the sEMG signals (Hargrove et al., 2007). In our study, for each of the three LDA classifiers, five time-domain (TD) features (Mean Absolute Value (MAV), Root Mean Square (RMS), Slope Sign Change (SSC), Waveform Length (WL) and Variance (σ^2^) were extracted from the corresponding channels of “raw” EMG data (Figure 3), in each analysis windows of 150 ms with an overlap of 100 ms (Smith et al., 2011). Since the LDA classifiers don't required the setting of internal parameters (Bellingegni et al., 2017), the training and test rely on a *two ways data split approach* (Ripley, 2007). Thus, the initial dataset was divided in this way: the TrainingSet (TR) contains 70% of the data and the test set contains the remaining 30% of the data. The training of the classifiers was performed by using the Equations (4,5). The subset were iteratively filled trough a *random shuffle* in order to obtain a configuration with proportionated class number (Bellingegni et al., 2017). The downsampling step wasn't necessary because the features extraction avoided the generation of large-scale-dataset and guaranteed a short time for the training of the classifiers. In details, the Linear Discriminant Analysis (LDA) with features extraction is a binary supervised machine learning algorithm able to transform the features into a lower dimensional space, which maximizes the ratio of the between-class variance to the within-class variance. This guarantees the maximum class separability (Welling, 2005). The following decision function is used to discriminate between only two different classes and to assign class label 1 or 2 to unknown data:

(4) $$h_{\beta }(x)=\{\begin{array}{l} (\beta^{T}\cdot x+\beta_{0})\geq 0\to 1\\ (\beta^{T}\cdot x+\beta_{0})<0\to 2 \end{array}$$

where β and β_0_ are, respectively, the classification parameters vector and the bias term. In details, the classification parameters can be evaluated in this way:

(5) $$\{\begin{array}{l} \beta =\Sigma^{-1}\cdot (\mu_{1}-\mu_{2})\\ \beta_{0}=-\beta^{T}\cdot \left(\frac{\mu_{1}+\mu_{2}}{2}\right)+\ln\left(\frac{\Pi_{1}}{\Pi_{2}}\right) \end{array}$$

where Σ is the pooled covariance matrix, μ_1_, μ_2_, Π_1_, Π_2_ are the mean vectors and the prior probabilities of class 1 and 2, respectively. Since LDA is a binary algorithm a *one vs. all* approach was implemented to solve the multi-class classification problem. The class label (c) is predicted as following:

(6) $$h_{\beta }(x)=\max_{c}\left({}_{c}\beta^{T}\cdot x{+}_{c}\beta_{0}\right)and\{\begin{array}{l} {}_{c}\beta =\Sigma^{-1}\cdot (\mu_{c})\\ {}_{c}\beta_{0}={-}_{c}\beta^{T}\cdot \left(\frac{\mu_{c}}{2}\right)+\ln\left(\Pi_{c}\right) \end{array}$$

where _*c*_β and _*c*_β_0_ are the classification parameters vector and the bias term of c class, respectively. An *ad hoc* developed software was implemented in Matlab for the construction of the three LDA classifiers.

The performance were evaluated through F1Score values and a Wilcoxon Signed-Rank test at *p* < 0.005 had been employed for comparing NLR and LDA classifiers in common datasets (Ortiz-Catalan et al., 2014). The LDA were trained and tested at 1KHz (without downsampling step) and for this reason the NLR model was evaluated considering the F1score on GS for the comparative analysis of the performance.

### 2.4. Experimental Setup

**Figure 5:**
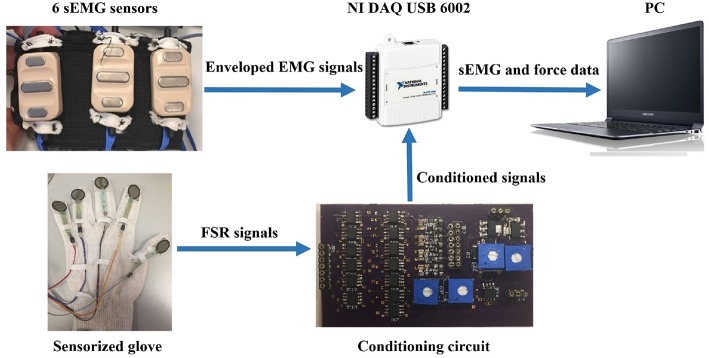
The experimental setup was composed by: (i) a sEMG elastic bracelet, (ii) NI DAQ USB 6002, (iii) a conditioning circuit, and (iv) glove equipped with Force Sensitive Resistors (FSR), Model 402 by Interlink Electronics.

Thirty-one healthy participants (age: 28 ± 7.6 years) were involved in the experiments. Six commercial active sEMG sensors (Ottobock 13*E*200 = 50, 27*mmX*18*mmX*9.5*mm*) were equidistantly fixed on an elastic adjustable bracelet and then were placed on the forearm of the able-bodied subjects in order to acquire sEMG signals (Figure 5).

The bracelet was located about 5*cm* below the subjects elbow, in line with the positioning of the electrodes, commonly used to control a prosthetic hand (Riillo et al., 2014). This type of electrodes output an enveloped signal of the “raw” signal (after amplification, filtering and rectification). The number of sEMG sensors was chosen equal to six because it was considered the highest number that was possible to place into the socket (Riillo et al., 2014). Moreover, it allowed to reduce the data dimensionality and complexity (Bellingegni et al., 2017). The EMG sensors operated in the range 0−5*V* with a bandwidth of 90−450*Hz* and a common rejection ratio higher than 100*dB*.

Five Force Sensitive Resistors (FSR), Model 402 by Interlink Electronics, were placed on a glove to verify the effective forces executed by the subjects. The relationship between the FSR voltage value *V* and the force value *F* was established with a statistically characterization as explained in Romeo et al. (2015). The relation between voltage and force is described trough the following mathematical expression:

(7) $$\begin{array}{r} \;F=p_{1}V^{5}+p_{2}V^{4}+p_{3}V^{3}+p_{4}V^{2}+p_{5}V+p_{6} \end{array}$$

obtained with the polynomial model:

(8) $$\begin{array}{r} y=\overset{n+1}{\underset{i=1}{\sum }}p_{i}x^{\left(n+1-i\right)} \end{array}$$

where *n*+1 represents the number of fitting coefficients, while *n* (1 ≤ *n* ≤ 9) is the degree of the polynomial. The Anderson loop was used as signal conditioning circuit (Anderson, 1998).

The EMG and force data were simultaneously acquired at 1 KHz using a suitable software on Labview platform by DAQ USB 6002 device. The PC (Samsung Intel(R) Core (TM) i7-4500U CPU @ 1.80 GHz 2.40 GHz) and DAQ communicated by means of an USB port.

**Figure 6:**
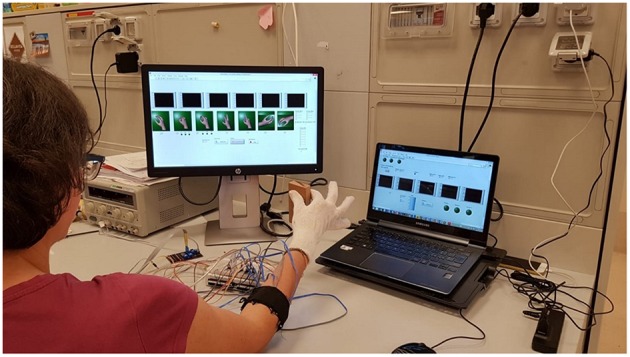
Subject positioning and data acquisition during experimental validation of the proposed approach. The subject was sitting in a comfortable chair in front of a PC monitor and was asked to perform six repetitions of each hand/wrist gesture. The subject performed “Spherical” and “Tip” gestures during the grasping of a rectangular object and executed three force levels. Written informed consent for the publication of this image was obtained.

The subject was sitting in front of a monitor (Figure 6) and was asked to perform the following seven hand gestures: Rest (hand relax), Spherical (hand with all fingers closed), Tip (hand with thumb and finger touching as if picking a small object), Platform (hand completely open and stretched), Point (hand with all fingers closed except for the index finger), Wrist Supination and Wrist Pronation. The participants were asked to produce each of these gestures for six times and hold it for 2 *s* with an interval of rest state about 2 *s* between each repetition.

In a initial phase before the training, each subject was asked to produce maximum muscle contractions in order to perform the highest peak of force, while grasping a stiff object of rectangular shape (weight 66 *g*, dimensions 50 × 100 × 17 *mm*) with “Spherical” and “Tip” grasps. The object was used also during the training session.

Three force thresholds were established at 30% (low), 60% (medium), and 90% (high) of the sum of all force contributions recorded from FSR sensors. Three force bands were defined as follows to reduce the difficult to perform a punctual value of force: the low level was fixed between the ±15% of the lowest threshold (i.e., 30%), the medium level was fixed as ±15% of the medium threshold (i.e., 60%), while the high level starts from −15% of the highest threshold (i.e., 90%) and continued until the maximum value. These bands were used to give a visual feedback to the subject during the recording of “Spherical” and “Tip” gestures.

## 3. Results

The results of the “hand/wrist gestures classifier” are reported in Table 1 in terms of the average accuracy and F1Score for NLR and LDA algorithms. The results of LDA classifiers with time domain features extraction were obtained with data sampled at 1 KHz (without downsampling). Thus, for the comparative analysis, we reported the results of NLR classifiers tested on “GS” because they represent the behavior of the classifiers when data sampled at 1 KHz are provided as input (Bellingegni et al., 2017). To this purpose, the Wilcoxon Signed-Rank test applied to the F1Score values was performed with significance threshold set to 0.05. Average classification accuracy for the NLR “hand/wrist gestures classifier,” the NLR “Spherical force classifiers” and “Tip force classifiers” are respectively equals to 98.78, 98.80, and 96.09%. The LDA “hand/wrist gestures classifier” reaches an average classification accuracy equals to 95.41%, while the LDA “Spherical force classifiers” and “Tip force classifiers” show an average classification value of 98.74 and 97.60%, respectively.

**Table 1:** Mean value and standard deviation of F1Score and Accuracy of the “hand/wrist gestures classifier” calculated for 31 healthy subjects with NLR and LDA algorithms.

**Hand/Wrist gestures classifier**								
	**NLR classifier**				**LDA classifier**			
	**F1_Score**		**Accuracy**		**F1_Score**		**Accuracy**	
**Classes**	**Mean (%)**	**Dev_std**	**Mean (%)**	**Dev_std**	**Mean (%)**	**Dev_std**	**Mean (%)**	**Dev_std**
Rest	98.25	4.05	99.50	1.24	97.62	4.31	99.05	3.55
Spherical	95.63	6.22	98.71	1.94	94.28	7.58	93.89	7.76
Tip	95.56	4.93	98.69	1.55	94.25	6.14	93.68	7.56
Platform	95.97	6.58	98.86	1.84	94.60	6.32	95.96	5.25
Point	92.69	9.25	97.63	3.58	93.52	6.02	92.38	7.67
Wrist supination	95.70	6.70	98.64	2.26	95.57	6.18	95.27	7.36
Wrist pronation	98.20	4.93	99.41	1.7	98	3.53	97.66	4.41

*The classification performance for the NLR classifiers are evaluated on “GS,” while LDA classifiers are tested on “TS,” with data sampled at 1 KHz (without downsampling)*.

The results of the two force classifiers, “Spherical force classifier” and “Tip force classifier” are shown, respectively, in Tables 2, 3, in terms of the average F1Score and accuracy for the NLR and LDA classifiers. The average classification accuracy of the NLR “Spherical force classifier” is 98.35% for the low force level, 98.25% for the medium force level and 99.80% for the high force level.The LDA “Spherical force classifier” shows an average classification accuracy of 98.7% for the low force level, 98.05% for the medium force level and 99.48% for the high force level. The average classification accuracy of the NLR “Tip force classifier” is 94.36% for the low force level, 94.46% for the medium force level and 99.26% for the high force level. The LDA “Tip force classifier" shows an average classification accuracy of 97.79% for the low force level, 96.36% for the medium force level and 98.66% for the high force level.

**Table 2:** Mean value and standard deviation of F1Score and Accuracy of the “Spherical force classifier” calculated for 31 healthy subjects with NLR and LDA algorithms.

**Spherical force classifier**								
	**NLR classifier**				**LDA classifier**			
**Classes**	**F1_Score**		**Accuracy**		**F1_Score**		**Accuracy**	
	**Mean (%)**	**Dev_std**	**Mean (%)**	**Dev_std**	**Mean (%)**	**Dev_std**	**Mean (%)**	**Dev_std**
Low	97.49	4.84	98.35	3.13	98.49	2.62	98.7	2.76
Medium	97.43	4.21	98.25	2.86	98.43	2.46	98.05	3.35
High	99.69	1.2	99.80	0.78	99.47	1.33	99.48	1.81

*The classification performance for the NLR classifiers are evaluated on “GS,” while LDA classifiers are tested on “TS” with data sampled at 1 KHz (without downsampling)*.

**Table 3:** Mean value and standard deviation of F1Score and Accuracy of the “Tip force classifier” calculated for 31 healthy subjects with NLR and LDA algorithms.

**Tip force classifier**								
	**NLR classifier**				**LDA classifier**			
**Classes**	**F1_Score**		**Accuracy**		**F1_Score**		**Accuracy**	
	**Mean (%)**	**Dev_std**	**Mean (%)**	**Dev_std**	**Mean (%)**	**Dev_std**	**Mean (%)**	**Dev_std**
Low	91.54	8.61	94.46	5.42	97.11	3.46	97.79	4.34
Medium	91.56	8.24	94.36	5.14	96.31	4.17	96.36	5.46
High	99.03	1.96	99.26	1.31	99.16	2.03	98.66	3.77

*The classification performance for the NLR classifiers are evaluated on “GS,” while LDA classifiers are tested on “TS,” with data sampled at 1 KHz (without downsampling)*.

**Figure 7:**
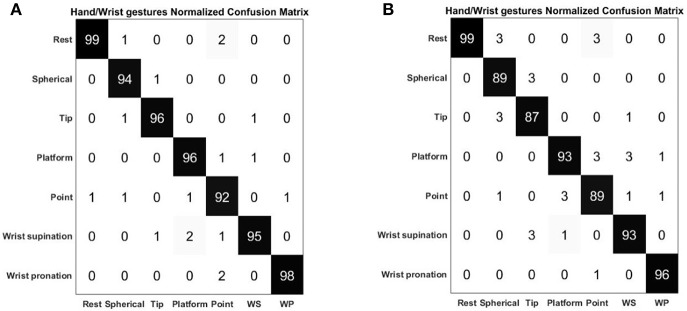
Normalized confusion matrix of the “hand/wrist gestures classifier” obtained with NLR algorithm **(A)** and LDA algorithm **(B)**. The confusion matrices are normalized with respect to the number of data belonging to the “GS” for the NLR classifier and to the “TS” for the LDA classifier. On the main diagonal the cardinality of the correct classifications is reported; in the top left dial and bottom right dial, the cardinality of the misclassified data related to the 7 output classes representing the hand gestures are reported.

**Figure 8:**
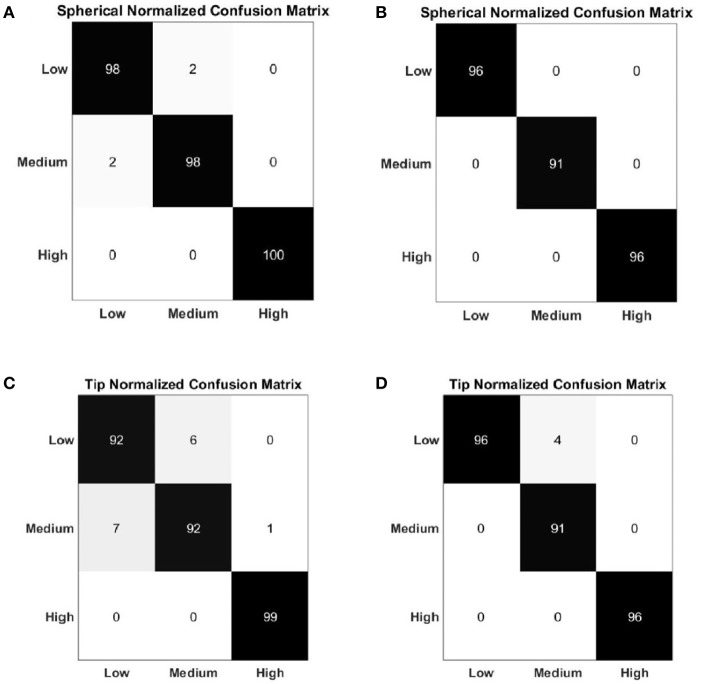
Normalized confusion matrix of the “Spherical force classifier” obtained with NLR algorithm **(A)** and LDA algorithm **(B)**. Normalized confusion matrix of the “Tip force classifier” obtained with NLR algorithm **(C)** and LDA algorithm **(D)**. The confusion matrices are normalized with respect to the number of data belonging to the “GS" for the NLR classifier and to the “TS” for the LDA classifier. The cardinality of the correct classifications is reported on the main diagonal; in the top left dial and bottom right dial, the cardinality of the misclassified data related to the 3 output classes that represented the force levels are reported.

**Figure 9:**
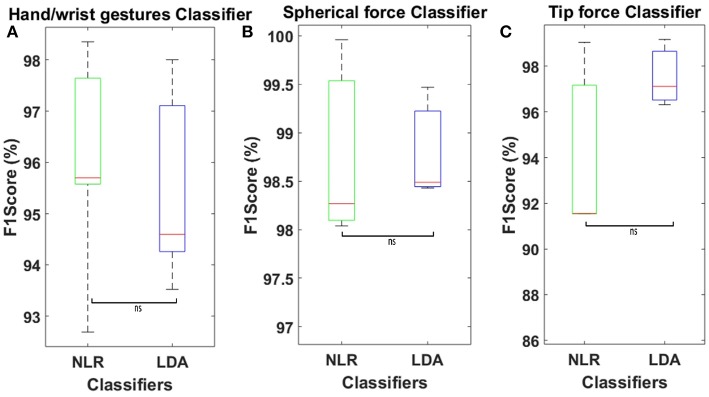
**(A)** Average F1Score values calculated on 30 healthy subjects using NLR “hand/wrist gestures classifier” algorithm, tested on “GS,” and LDA “hand/wrist gestures classifier” with 5 time domain features, tested on “TS.” **(B)** Average F1Score values calculated on 30 healthy subjects using NLR “Spherical force classifier” algorithm, tested on “GS,” and LDA “Spherical force classifier” with 5 time domain features, tested on “TS.” **(C)** Average F1Score values calculated on 30 healthy subjects using NLR “Tip force classifier” algorithm, tested on “GS,” and LDA “Tip force classifier” with 5 time domain features, tested on “TS.” Statistical non-significance is indicated by “ns”.

Figure 7 shows the average confusion matrix when testing the NLR and LDA “hand/wrist gestures classifier” on “GS” and “TS,” respectively. In details, Figure 7A reports the normalized confusion matrix for the NLR “hand/wrist gestures classifier,” while Figure 7B is related to the LDA “hand/wrist gestures classifier.” Figure 8 shows the average confusion matrices when testing the NLR “Spherical force and Tip force classifiers” on the “GS” (Figures 8A,C) and the LDA “Spherical force and Tip force classifiers” on “TS” (Figures 8B,D). As shown in Figure 9 the NLR and LDA “hand/wrist gestures classifier” were able to identify seven hand gestures with an average F1Score of 96.01% and 95.41% respectively (Figure 9A). The “Spherical force classifier” identified the force level reaching an average F1 score of 98.75 and 98.79% with NLR and LDA classifiers, respectively (Figure 9B). The “Tip force classifier” was able to define the force level with an average F1 score of 94.04 and 97.53% with NLR and LDA classifiers, respectively (Figure 9C). The Wilcoxon Signed-Rank test applied to the F1Score values points out no statistically significant difference (‘ns") between NLR and LDA algorithms (at *p* < 0.05).

**Figure 10:**
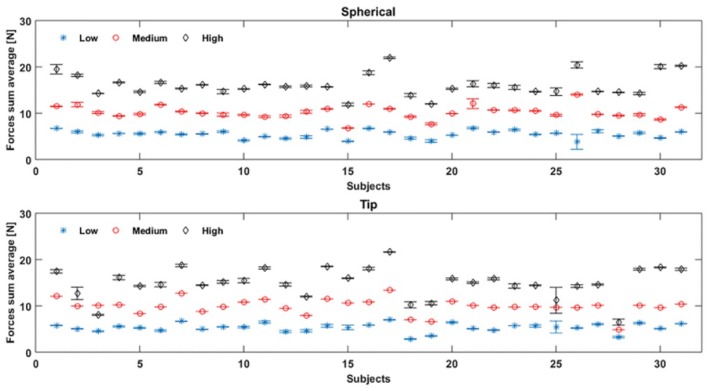
Force sum average values are obtained, by FSR measurements, for 31 healthy subjects during, respectively, the “Spherical” and “Tip” gestures, performed six times: the blue, red and black values represent the mean value and standard deviation of respectively low, medium, and high force values performed by each subject.

In Figure 10 the average values of the sum of all the FSR measurements for 31 healthy subjects are showed. The misclassification error rates are presented in Tables 4, 5 for both the NLR and LDA “hand/wrist gestures classifier”. The NLR “hand/wrist gestures classifier” performed the highest misclassification errors (i.e., 9%) with “Point” class, while the LDA “hand/wrist gestures classifier” performed misclassification errors greater than 10% (i.e., 11, 12.7, and 11.3%) for the “Spherical, Tip and Point” classes, respectively. The NLR and LDA “Spherical force classifier” reached the maximum misclassification error (i.e., 3 and 8%, respectively) for the “Medium" force level. The “Tip NLR and LDA force classifier” presented the same maximum misclassification error (i.e., 8.5%) for the “Medium” force level.

**Table 4:** Misclassification error rates of the “hand/wrist gestures classifier" calculated with NLR and LDA algorithms.

**Misclassification error rates (%)**		
	**Hand/Wrist gestures classifier**	
**Classes**	**NLR classifier**	**LDA classifier**
Rest	1	1
Spherical	6	11
Tip	5	12.7
Platform	5	7
Point	9	11.3
Wrist Supination	5	7
Wrist Pronation	2	4

**Table 5:** Misclassification error rates of Spherical and Tip Force Classifier calculated with NLR and LDA algorithms.

**Misclassification error rates (%)**				
	**NLR classifier**		**LDA classifier**	
**Classes**	**Spherical force classifier**	**Tip force classifier**	**Spherical force classifier**	**Tip force classifier**
Low	2	8	4	4
Medium	3	8,5	8	8.5
High	1	2	4	4

## 4. Discussion

As shown in Table 1 and Figure 9 the NLR and LDA “hand/wrist gestures classifier" were able to identify seven hand gestures with an average F1Score of 96.01 and 95.41% respectively. The “Spherical force classifier” identified the force level reaching an average F1 score of 98.75 and 98.79% with NLR and LDA classifiers, respectively (Table 2). The “Tip force classifier" was able to define the force level with an average F1 score of 94.04 and 97.53% with NLR and LDA classifiers, respectively (Table 3). These results seem to be very promising if we consider that similar values of average F1Score have been achieved only for gesture classification (Duan et al., 2016; Bellingegni et al., 2017). The comparative analysis between NLR and LDA classifier, applied to F1Score values, reported no statistically significant difference (*p* < 0.05) between them.

Confusion matrices, reported in Figures 7, 8, confirmed the positive results of the accuracy parameter. The cardinality of the correct classifications on the main diagonal underlined the high classification accuracy even if some misclassified data out of the main diagonal suggested a bit minus performance of “Tip force classifier” respect to “Spherical force classifier.” This is due to the major difficulty encountered by few subjects to modulate between low and medium force levels during a Tip grasp. The high force levels were always well discriminated at 99% of average accuracy for both the NLR and LDA force classifiers. In Table 4, the LDA “hand/wrist gestures classifier” obtained a greater misclassification error rates than NLR “hand/wrist gestures classifier” ranging from 1% to maximum 12, 7% for discriminating seven hand/wrist gestures classes by using data including different muscular activations related to desired force levels. This may due to the fact that linear classifiers, with straight line or plane decision boundary, could not be the most appropriate method for a seven multi-class problem with features not linearly separable at all. In comparison to the results presented in Scheme and Englehart (2011), the misclassification error values, obtained for the “Spherical” and “Tip” classes with the LDA “hand/wrist gestures classifier,” were lower than 17% and, thus, it can be considered an effective result. Moreover, the misclassification error values, obtained for the “Spherical” and “Tip” classes with the NLR “hand/wrist gestures classifier” were, respectively, equals to 6 and 5% and these results can be considered positive for an usable system (<10%) (Scheme and Englehart, 2011). Finally, the misclassification error rates for the “Spherical and Tip force classifiers” are similar (Table 5), ranging from 1% to maximum 8, 5% for both the NLR and LDA classifiers.

Almost all healthy subjects were able to modulate the force levels and fall into the range displayed by the visual feedback, without generating high variance values, as shown in Figure 10. Fewer subjects difficulty reproduced the force values within the force intervals, despite the visual feedback as reference. For instance, in Figure 10, the subjects 25 and 3 were not able to well differentiate between medium and high force levels during Tip grasp (represented as red and black points), while subject 28 performed the three force levels too closed during Tip grasp. This depended on the subject's difficulty to maintain the applied force within the force intervals.

These results are also more appreciable if we take into account that NLR, used for the classification of both hand/wrist gestures and force levels, was trained and tested using only raw scaled sEMG signals as input features. On the other hand, the LDA algorithm employed the minimum number of classification parameters and computational burden. However, the use of time domain features extraction based on time windowing, make the class evaluation time equals to the window shift and the system delay approximates to the time window length (Bellingegni et al., 2017). Furthermore, the same number of sensors were adopted to classify seven gesture classes respect to the previous five (Bellingegni et al., 2017) and to identify three levels of force during the execution of “Spherical" and “Tip" grasps. The proposed hierarchical classification architecture permitted to decode the user's motion intention and desired force levels with high reliability. Despite the proposed PR approach was tested only on healthy subjects, the reported results are promising for future developments on trans-radial amputees. The proposed hierarchical pattern recognition approach has obtained effective results with both NLR and LDA algorithms that have been demonstrated to be suitable for discriminating both hand/wrist gestures and force levels applied during grasping tasks. Moreover online performance will be evaluated for controlling a multi-functional prosthetic device.

## 5. Conclusion

In this study a hierarchical classification approach was developed and tested to discriminate both hand/wrist gestures and force levels applied during grasping tasks. The proposed PR system, implemented with both NLR and LDA classifiers, was tested on 31 healthy subjects by using 6 commercial sEMG sensors and five FSR placed on a glove. The method employed three different classifiers to discriminate both desired gestures and forces. To this purpose, the NLR and LDA algorithms were adopted for implementing the hierarchical classification approach and a comparative analysis among the performance of these two algorithms was done. The statistical analysis based on the Wilcoxon Signed-Rank test, applied to the F1Score values, revealed no statistically significant difference between NLR and LDA. The NLR classifiers exhibited excellent results in terms of accuracy both for gestures (i.e., 98.78%) and forces (Spherical 98.80%, Tip 96.09%). In particular, the force classifiers were able to robustly discriminate the same class of movement performed at different muscle contractions because they were trained with data containing the modulation of different force levels. Also the LDA classifiers achieved effective results in terms of accuracy both for gestures (i.e., 95.41%) and forces (Spherical 98.74%, Tip 97.60%). The misclassification errors of the NLR classifiers was limited to a maximum values of 9% for the “hand/wrist gestures classifier,” 3% for the “Spherical force classifier” and 8.5% for the “Tip force classifier.” On the other hand, the misclassification errors of the LDA classifiers reached the maximum values at 12, 7% for the “hand/wrist gestures classifier,” 4% for the “Spherical force classifier" and 8.5% for the “Tip force classifier.” In particular, the results of misclassification values obtained by the NLR and LDA “hand/wrist gestures classifier” for the “Spherical” and “Tip” classes, were particularly noteworthy and promising. Based on these outcomes, a new potential strategy should be introduced for mitigating the effect of different exerted forces within a given movement class. Another innovative contribution is represented by the use of FSM theory for the management of three classifiers. This control strategy avoids to face a more seven multi-class problem using a single classifier and make the system controllability less complex by activating the force classifiers only when the “hand/wrist gestures classifier” returns an output class belonging to a closure hand gesture. This classification approach, implemented both with NLR and LDA algorithms, have obtained positive results and seems to be very promising for identifying simultaneously desired gestures and force levels.

In conclusion, the proposed method allowed to extract from EMG signals all the valuable information regarding not only muscle contractions related to hand/wrist motions but also the changes of muscle activation patterns depending on the influence of different force levels. This approach will allow to improve the performance of the currently adopted prosthesis EMG control architectures thanks to the possibility to manage desired gestures and force levels in a more natural way. The ultimate goal will be to produce an intuitive controlled hand prosthesis integrating force regulation. Although the type of the recruited subjects did not allow to verify the performance in a real application scenario, this study permitted to provide a general indication about the performance of the proposed approach. Future works will be focused on the validation of the presented method on trans-radial amputees controlling multi-fingered hand prostheses. Moreover, online performance will be evaluated in real application scenario. After reaching an advanced grade of real time accuracy, an embedding version of this classification system will be developed to control a prosthetic device. Measures of system robustness and reliability will be performed testing the proposed approach during the control of prosthetic devices. Advanced control strategies (Ciancio et al., 2015; Barone et al., 2016) will be adopted to allow force regulation and slippage management during grasping (Cordella et al., 2016).

## Ethics Statement

The experimental protocol was approved by the local Ethical committee (Comitato Etico Université; Campus Biomedico di Roma) and complied with the Declaration of Helsinki. All subjects gave written informed consent in accordance with the Declaration of Helsinki.

## Author Contributions

FL analyzed the literature, designed the proposed approach, acquired, and analyzed the experimental data and wrote the paper. CG contributed to the design of the proposed approach, to the analysis of the experimental data and wrote the paper. AC contributed to the design of the proposed approach and of the experimental setup, wrote the paper, and supervised the study. EGr contributed to the design of pattern recognition algorithm and contributed to the manuscript writing. AD contributed to the manuscript writing. RS contributed to the manuscript writing. EGu contributed to the manuscript writing. LZ designed the paper and supervised the study. All the authors read and approved the manuscript.

### Conflict of Interest Statement

The authors declare that the research was conducted in the absence of any commercial or financial relationships that could be construed as a potential conflict of interest.
